# Measuring follow-up time in routinely-collected health datasets: Challenges and solutions

**DOI:** 10.1371/journal.pone.0228545

**Published:** 2020-02-11

**Authors:** Daniel Thayer, Arfon Rees, Jon Kennedy, Huw Collins, Dan Harris, Julian Halcox, Luca Ruschetti, Richard Noyce, Caroline Brooks

**Affiliations:** 1 SAIL Databank, Swansea University Medical School, Swansea, United Kingdom; 2 Swansea University Medical School, Swansea, United Kingdom; 3 Abertawe Bro Morgannwg University Health Board, Swansea, United Kingdom; University of Toronto, CANADA

## Abstract

A key requirement for longitudinal studies using routinely-collected health data is to be able to measure what individuals are present in the datasets used, and over what time period. Individuals can enter and leave the covered population of administrative datasets for a variety of reasons, including both life events and characteristics of the datasets themselves. An automated, customizable method of determining individuals’ presence was developed for the primary care dataset in Swansea University’s SAIL Databank. The primary care dataset covers only a portion of Wales, with 76% of practices participating. The start and end date of the data varies by practice. Additionally, individuals can change practices or leave Wales. To address these issues, a two step process was developed. First, the period for which each practice had data available was calculated by measuring changes in the rate of events recorded over time. Second, the registration records for each individual were simplified. Anomalies such as short gaps and overlaps were resolved by applying a set of rules. The result of these two analyses was a cleaned set of records indicating start and end dates of available primary care data for each individual. Analysis of GP records showed that 91.0% of events occurred within periods calculated as having available data by the algorithm. 98.4% of those events were observed at the same practice of registration as that computed by the algorithm. A standardized method for solving this common problem has enabled faster development of studies using this data set. Using a rigorous, tested, standardized method of verifying presence in the study population will also positively influence the quality of research.

## Introduction

In observational studies, it is crucial to only include individuals who either experienced events or states of interest, or are at risk of experiencing them. To be at risk means not only that individuals have the possibility of (for example) contracting a particular disease, but also that this event would be recorded in the data being used if it were to happen [1]. Failure to correctly identify the population at risk within an analysis may lead to bias in a study. Likewise, it is critical to know which individuals are followed up, and for how long, when measuring outcomes [2].

Identifying those individuals who are at risk of experiencing particular events or states becomes increasingly complex when conducting health research using observational data sources [3,4]. There is often no simple answer to the question of when an individual is part of the population covered by a particular dataset. Numerous potential factors may affect individual presence for data recording: life events such as birth or death; an individual moving in and out of the covered geographic area; the beginning and end of the period when data is available; and limitations in the coverage of the dataset itself.

All of these factors must be accounted for when determining which individuals to study and measuring follow-up time. Simplistic methods of dealing with this problem are subject to bias. If presence in the data is measured only at a single point in time, there will be gradual attrition from the cohort as observations are recorded further from the point of measurement, skewing longitudinal results. Using events recorded in the data as an indicator of presence can introduce selection bias, since a health record with no events recorded is often a clinically valid outcome. For example, a woman who lived in the catchment area of a hospital for five years but never had a hospital admission is still part of the population of interest, because if an admission had occurred, she would have appeared in the records. Excluding such individuals from a study would introduce a bias towards those who have received care or treatment.

Datasets held by the Secure Anonymised Information Linkage (SAIL) Databank, a large repository of health data covering the Welsh population [5,6], exemplify these issues. In particular, the primary care GP dataset holds records for only some of the general practitioners (GPs) in Wales, as practices are the data owners and must individually agree to contribute their data to SAIL [7]. The time period for which data is available varies by practice, depending on when electronic recordkeeping began and how recently data was submitted. Furthermore, each individual has a potentially complicated history, with an arbitrary number of registrations with different GPs and moves in and out of Wales.

Data errors, quirks, and inconsistencies, an ever-present reality when working with routine data [8], add a final layer of complexity to the problem. Examples of such issues in the GP registration history in the Welsh Demographic Service (WDS) include duplicate rows, overlapping periods of registration with different practices (which is not possible according to NHS administrative rules), and multiple consecutive periods of registration with the same practice.

Properly accounting for all of these factors in order to identify when an individual is part of the population at risk for a particular study is a significant undertaking, and one that must be addressed by all studies using primary care data. We created and automated a procedure to determine individual presence within this dataset. This tool, which is adaptable to different research needs, is made available to all researchers working with the data, ensuring that all projects can benefit from a rigorous, tested, consistent solution to this problem.

## Methods

An individual’s availability for follow-up in a dataset is the intersection of two different factors: the coverage of the dataset (both in historical and organisational terms) and the individual’s history of when he or she was within that coverage area. There are two main challenges when measuring individual follow-up time in this dataset: identifying when each practice started recording data, and converting each individual’s potentially complex history into a simple measure of when they are present in the dataset. To account for these two challenges, a two-step algorithm was developed.

### Determining dataset coverage

The primary care dataset in SAIL covers all patients registered with practices that have agreed to share data. For each individual practice, the beginning of usable data is the date when the practice started using coded electronic records—which is not known. The end of usable data is the date the of the last data extract from that practice (usually within the last twelve months, but when a practice has closed or a particular extract failed to run, a greater lag time may be observed).

#### Possible methods of inferring the start date of electronic record keeping

In order to determine the start and end dates of data coverage, the data itself is examined for each practice. The most obvious way to do this, by finding the earliest and latest event recorded, turns out to be problematic. Practice systems allow an arbitrary value to be entered for the date of an event, resulting in some events receiving erroneous dates. Events are recorded improbably far in the past (1^st^ January, 1900 is a particularly popular date, almost certainly being applied to events of unknown date). Some events even have dates in the future. In addition to dates that reflect missing data or errors, events may be legitimately backdated when they are entered. For example, a GP may retrospectively code the date that an individual was first diagnosed with a disease.

Some research datasets include the date of recording in addition to the event date; for example, this is the case in the Clinical Practice Research Datalink (CPRD), a UK-wide primary care research database [9]. If this were present, it would serve as a more reliable method of ascertaining the period of available data, but it is currently not available in the SAIL GP dataset.

#### Measuring the start date based on event rates and a threshold

Measuring the volume of events recorded over time enabled estimation of the start date of electronic record keeping at each practice. When a practice is truly recording data electronically, we expect there to be a large volume of events recorded on a regular basis, while the volume of erroneous dates and events that are retrospectively recorded is relatively low by comparison.

In order to implement this, the data for each practice was divided into one-month periods. The number of individuals registered at each practice was counted on the 15^th^ day of each month and compared to the total number of events recorded in that month. This gave a rate of events recorded per person per month for each practice. These calculations were stored in an intermediate table, which only needs to be recalculated when the dataset is updated. A practice was required to have more than five people registered in a given month for that month’s data to be counted. This served to eliminate a few cases of apparent missing GP registration data. Note that the algorithm is based only on event volumes measured in aggregate at each practice. Whether an individual has events recorded is never used as a criterion for that individual being followed up in the data; to do so would introduce a bias against healthy individuals into the coverage measure.

For early versions of the algorithm, the monthly event rate was calculated as the average number of events per person. However, use of the average was susceptible to skewing caused by concentration of events on particular days: for data recorded retrospectively, events tend to be recorded on January first of the year, if only the year is known; or the first of the month, if only the month and year are known. This has an outsized impact on early periods where much of the data has been retrospectively recorded, leading to spikes on these days. This, in turn, leads the measured event rate for January to be unusually high. In addition, there may be single days where are a large of events end up being recorded for administrative or technical reasons. To address this, the median events per person recorded on weekdays were used instead.

This event rate was normalized on a per-practice basis with reference to the rate of event recording during a period where data was known to be available. The year 2009, which is believed to be after the date that electronic recording started at all practices, was used as the reference period when available; for a few practices that didn’t have registration data as early as 2009, the first complete year (defined as the first year with at least 100 patient registration records starting before January first of that year) was used as the reference period. All other rates were calculated relative to the year that was applied as the reference period.

**Fig 1:**
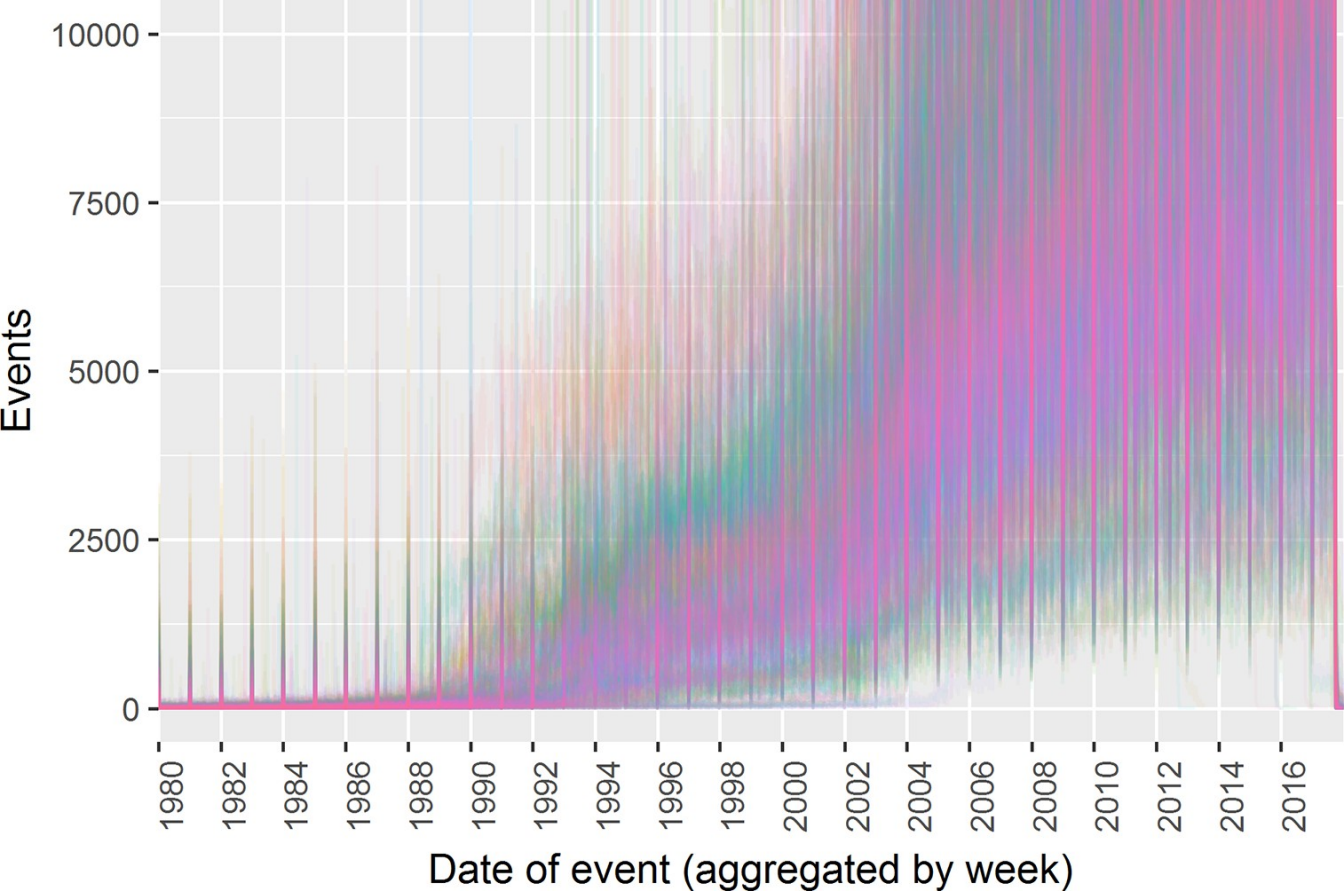
Event volume over time by GP practice.

Fig 1 shows the historic rate of event recording for all practices. Each line represents the rate of events recorded per registered patient over time at a single practices; a few practices appear to increase their event recording suddenly at a given date, but many increase gradually. No clear pattern emerges that would suggest an obvious threshold or algorithm for identifying the start of recording. This illustrates the challenge of identifying a clear start date for electronic data recording.

The start date of data for each practice are determined by the application of a threshold to the normalized event rate. Because no clear picture emerges of what a “normal” rate would be for electronic recording, as shown in Fig 1, the procedure allows the end user to specify a threshold, enabling researchers to use different thresholds depending on their requirements. Since the date on which data was extracted is known, usually the day before that date is taken at the end date; however, the end date is also checked using the rate of event recording, and if the algorithm finds that data was not being recorded up to the end, the actual end date of recorded data is taken (this may perhaps occur in scenarios such as a practice closing and then later extracting data).

#### Determining individual presence within the dataset

Once we have identified the start and end date for the data at each practice, we then go on to check what individuals are registered at these practices during the period of available data. A history of individuals’ GP practice registrations is available within the WDS [10]. Registration records are available for all of Wales, not just those practices submitting to SAIL, since WDS is held centrally by the NHS Wales Informatics Service (NWIS). One individual may have many records in this dataset: individuals may move in and out of Wales, move around within Wales, and register with different practices while living at the same location. There are also sometimes additional records due to consecutive records at the same practice (the reasons for which are not clear), as well as occasional errors such as duplicated or overlapping records. Writing code that considers all of these issues is complex and time consuming; hence we wished to provide a simplified record that provides the information that is typically needed to measure study follow-up.

#### Potential biases in follow-up measures

Individual follow-up is measured based only on registration records, not recorded clinical information. Using the actual events recorded to determine follow-up would bias the resulting population toward those who have used services. The goal is to obtain the population that would appear in the data if they visited the GP; someone who never went to the doctor but would have appeared in the data had they gone, should not be excluded on the basis of having no events in the data.

Even the use of registration records does not completely eliminate bias, since registration with a GP is also likely to be at least somewhat correlated with receiving medical care, and thus the coverage measure can’t be made completely independent of individual health state. One particular concern is that individuals may move out of Wales and fail to de-register with their Welsh GP, thus making it appear that they are still present in the data, when they are in fact lost to follow-up. Patients who have moved outside of a practice area are supposed to be eventually removed from the practice list [11], but this process takes time, during which the patient will still be marked as registered with the practice. There is no single solution to mitigate the impact of such an issue, and each research study needs to take care to consider and address potential biases that may be caused by this and other limitations of the data.

#### Parameters affecting aggregation for different follow-up measures

Each individual’s registration history was simplified based on a customizable set of aggregation rules. Two adjacent records are considered combinable based on the two variables that set the output grouping: group_on_sail_data and group_on_practice. If group_on_sail_data is set to 1, registrations with SAIL data will never be combined with registrations that don’t have SAIL data. If group_on_practice is set to 1, records from different practices will never be combined. As shown in Table 1, these two variables work together, allowing various combinations with different uses:

**Table 1:** Effects of combinations of two grouping parameters.

group_on_sail_data	group_on_practice	Results Produced
0	0	Periods in which people are continuously registered with any Welsh GP.
1	0	Periods in which people have GP data available on SAIL.
0	1	Cleaned version of GP registration history.
1	1	Cleaned GP registration history, with flag indicating whether each record has SAIL data.

A given registration record may overlap the start or end date of the available data at the practice, and therefore, data may be available for only part of the registration period. Where this occurs, and group_on_sail_data is 1, the registration is split into two (or three) new records. This splitting is done first, before any of the combining logic described above.

#### Resolving conflicts

There are a number of erroneous cases of an individual having multiple registration records covering the same period. While these are rare situations, when they do occur they can cause problems such as duplication when querying the data. So, in all cases the procedure resolves these so that individuals are only registered with a single practice at any given point. In order to determine which record should be preferred in particular overlapping scenarios, analysis was conducted to check which records were more likely to have events. Analyses of the data as it existed in June 2013 were conducted to identify the best choices to make to resolve conflicts.

*Nested records*. Nested registration records are resolved by selecting the first (outer) record. These cases are rare (55 observed where both practices are in SAIL), and in most cases the second registration is for a short period (27 of the 55 were for a single day, and only 18 were for 90 days or more). In the cases where the second registration is >90 days, the second registration is somewhat more likely to have events. However, because the observed numbers are too small to reason about which record is “correct”, and because taking the second registration would add complexity and fragment the data, the first registration is selected in all cases.

*Overlapping records*. If two records overlap, the first record is shortened so that it ends just before the second record starts. There were 182 cases of overlap in which both GP practices were in SAIL. In only 5 of these cases (3%) did the first registration have more events in the GP data during the overlap period. In 94 cases (52%) the second registration had more events. Thus, it seems preferable to always use the second registration for the overlapping period.

*Duplicate periods*. There were 2931 cases of individuals registered to two different practices over identical time periods. In this case, there is no way to programmatically identify which is the correct record. In order to select which record to keep, following rules are applied (in order):

1. Prefer records with GP data on SAIL.
2. Prefer records with lower encrypted practice ID (an arbitrary choice, but it ensures that the algorithm will be deterministic).

These preferences are always used, even though they may not be relevant to the output, depending on what type of output is requested from the procedure. This is so that the same records will always be chosen, regardless of what options are used.

*Short gaps between records*. Sometimes, there are short gaps between registration records. More than 12,000 pairs of consecutive records for the same individual have a gap of between 2 and 30 days (though this is only 0.16% of all record pairs). Individuals are unlikely to move outside Wales for a few days or a few weeks, then move back. The gaps are more likely to be a delay in registering with a new GP after moving to a new area or de-registering with another doctor. Based on this assumption, an individual is most likely part of the population covered by the primary care data during this gap: had treatment from a GP been required during the period, they would have registered with a GP earlier and appeared in the dataset. Thus, during these gaps, the individual was assumed to be present in the data for the second practice. The length of such gaps to close is customisable according to the needs of each research project, with the default value being 30 days.

#### Missing historical data

A specific issue with the data provision to SAIL was discovered, whereby certain patients who had deceased or de-registered from a practice had their data excluded. When this issue was identified, the procedure was modified to exclude periods of missing data for individuals from the measured coverage.

### Implementation and testing

The solution was implemented as two DB2 SQL stored procedures. The first, run every time the GP dataset is updated, calculates the monthly event rates per practice. The second is used by researchers to produce customizable records of coverage for the individuals in their studies.

A set of unit tests of the behaviour of the procedures was created and run. These tests were automated, so that they can be easily repeated in the future if the code is modified.

### Analyses to show the impact of follow-up measures

We conducted two simple analyses using several different measures of follow-up time in order to show the potential impact of how follow-up is measured on research. Atrial fibrillation (AF) is a common heart condition characterised by heart rate abnormalities. Individuals with AF are at increased risk of stroke; they are often treated with blood-thinning medication such as Warfarin.

We identified individuals diagnosed with AF in primary care, then followed up for ten years, measuring the likelihood of receiving a Warfarin prescription and the incidence of stroke in each year. Patients who had died were excluded. This was repeated for a number of follow-up methods, as shown in Table 2:

**Table 2:** Overview of different follow-up requirements in example analysis.

Followup Requirement	Description
None	Any individual with a diagnosis of AF was included in the study; no further restrictions.
Present at diagnosis	An individual was required to be registered with a practice submitting data to SAIL at diagnosis.
Present at start of year	An individual was required to be registered with a practice submitting data to SAIL at the start of the year being measured.
Present entire year	An individual was required to have data continuously available in SAIL for the entire year.
Present entire 10 years	An individual was required to have data continuously available in SAIL for the entire ten years.

## Results and discussion

The primary care dataset contains roughly 2.4 billion events, and 99.6% are linkable to individuals in WDS with GP registration records available. When the coverage procedure is run using a 10% event rate threshold, 91.0% of all linkable events in the GP dataset occur during a period when the algorithm considers the individual to have data available in the dataset. 98.4% of these events occur at the same practice with which the algorithm considers the individual to be registered. After excluding data with old dates that is likely to be the result of retrospective recording (before 2000) and data for individuals not found in the WDS, 94.3% of the records that remain are within periods when the individual is considered present. Out of these events, the event occurs at the same practice that the individual was marked as registered at 98.5% of the time.

**Fig 2:**
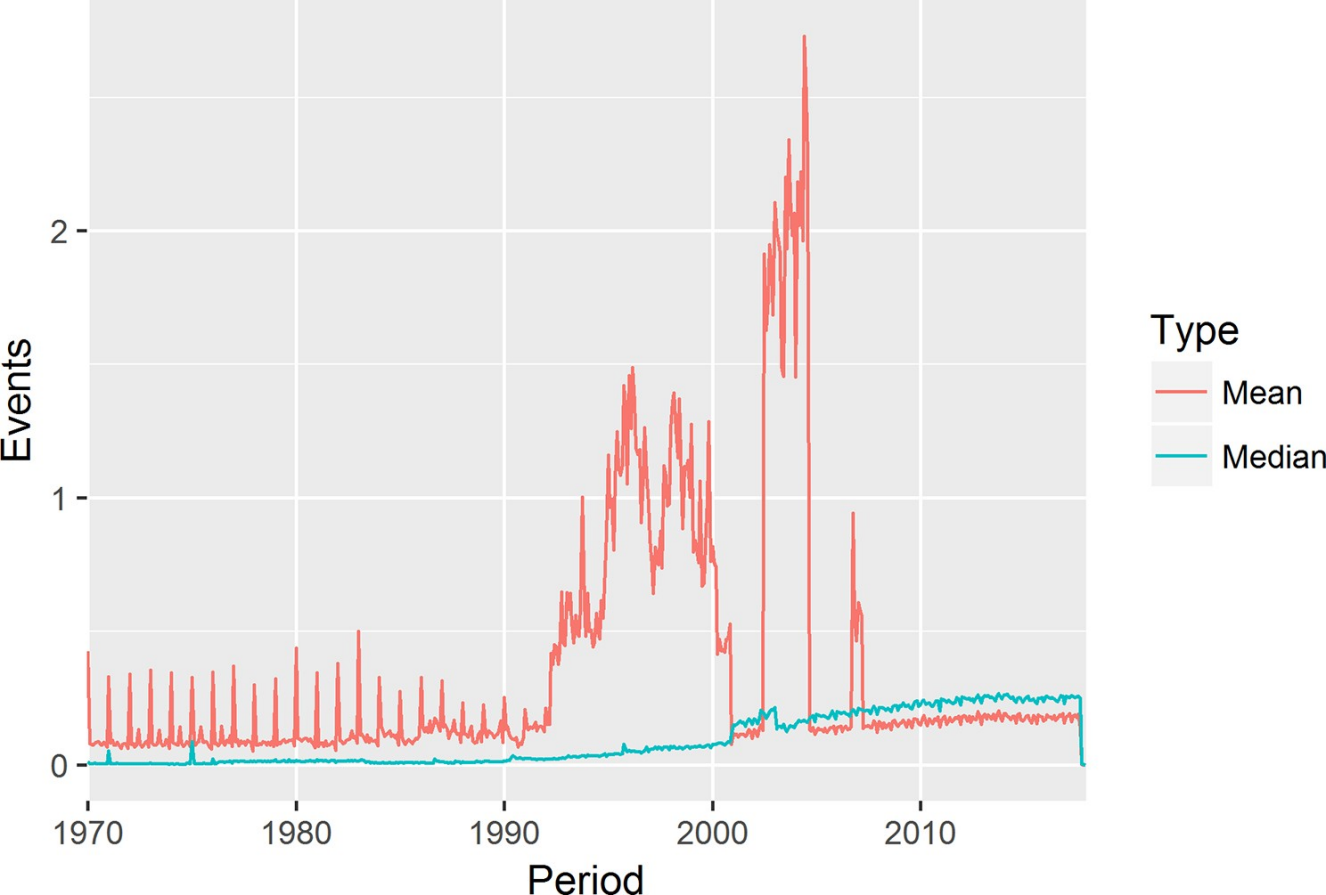
Monthly event recording rates: Mean vs. median.

The use of the median monthly weekday events to calculate practice event rates results in a smoother increase of event rates over time, as compared to the mean, as shown in Fig 2.

### Performance

Performance of the procedure will vary based on the hardware environment, data size, database load, etc. The SAIL Databank is held on an IBM DB2 database (version 11.1 LUW), hosted on an x86 Centos 7 cluster, consisting of five nodes with 24 cores and 254 GB of RAM each. In ten test runs during normal working hours, the procedure took an average of 9 minutes 10 seconds (minimum 6:44; maximum 10:08).

### Validation

Unit testing was conducted to verify that the procedure performs as it was designed. The test results show that individual registration records are correctly derived from the Welsh Demographic service and practice coverage matches periods when appropriate volumes of data are being recorded in practice records. The full set of tests can be found in the project repository in Github (https://github.com/DSThayer/gp-coverage-tool).

### Impact of different coverage measures

The simple analysis of Warfarin prescriptions and stroke outcomes in AF patients demonstrates the impact that the choice of coverage measure can have on results. Even at the time of diagnosis, when the presence of a diagnosis in the primary care record suggests that the individual is very likely to be followed up in the data, the use of a follow-up requirement has a major impact on the measured rate of Warfarin prescription. Only 46.2% of the total cohort diagnosed with AF has a Warfarin prescription recorded in the year following diagnosis; simply requiring that the individual is registered with a practice with primary data available on the data of diagnosis increases the measured prescription rate to 50.9%, while requiring data to be available for the entire year increases this number only very slightly to 51.0%. Among individuals with data available for a full 10 years, the rate is even higher at 52.0%.

**Fig 3:**
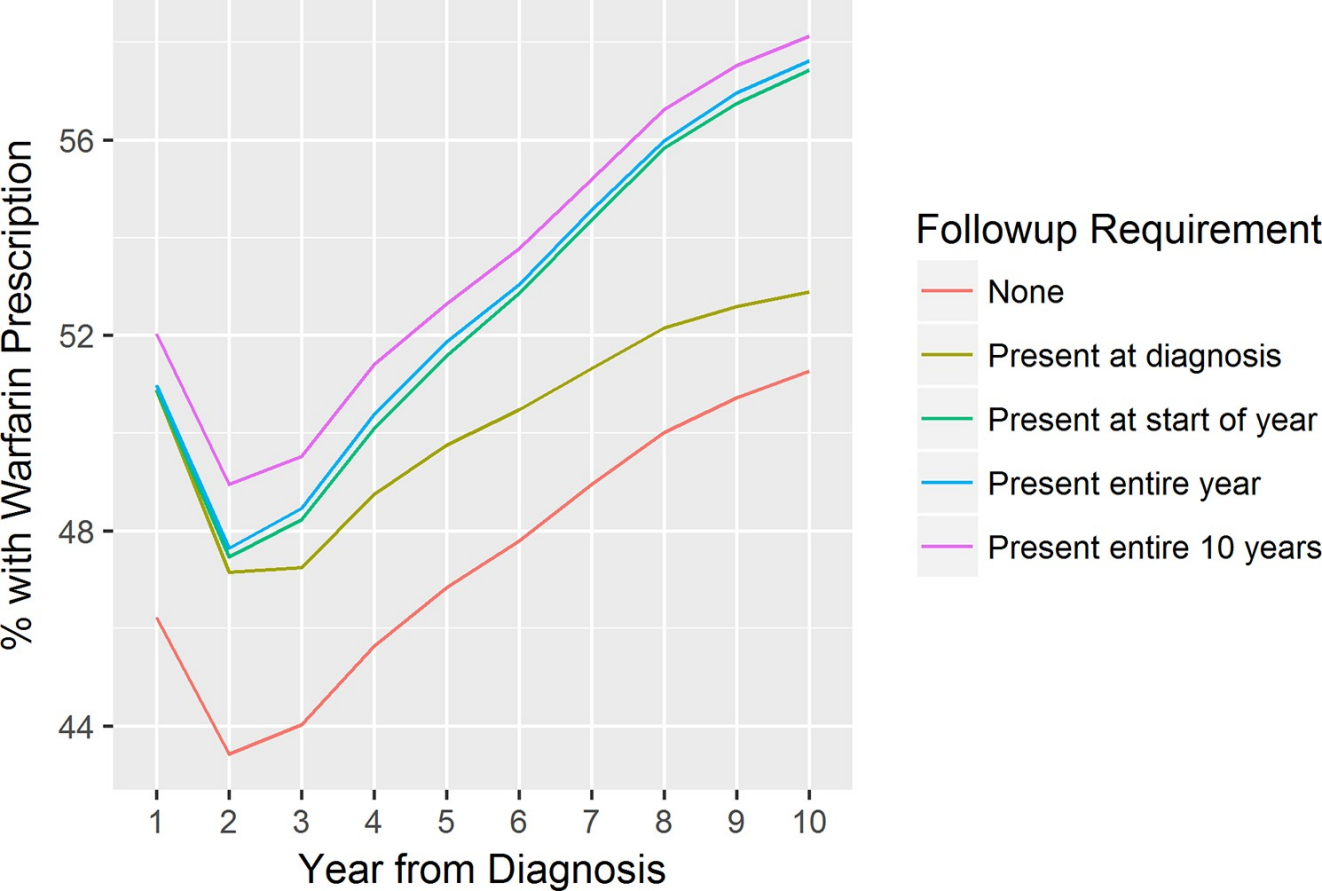
Measured rate of warfarin prescription by year from AF diagnosis.

**Fig 4:**
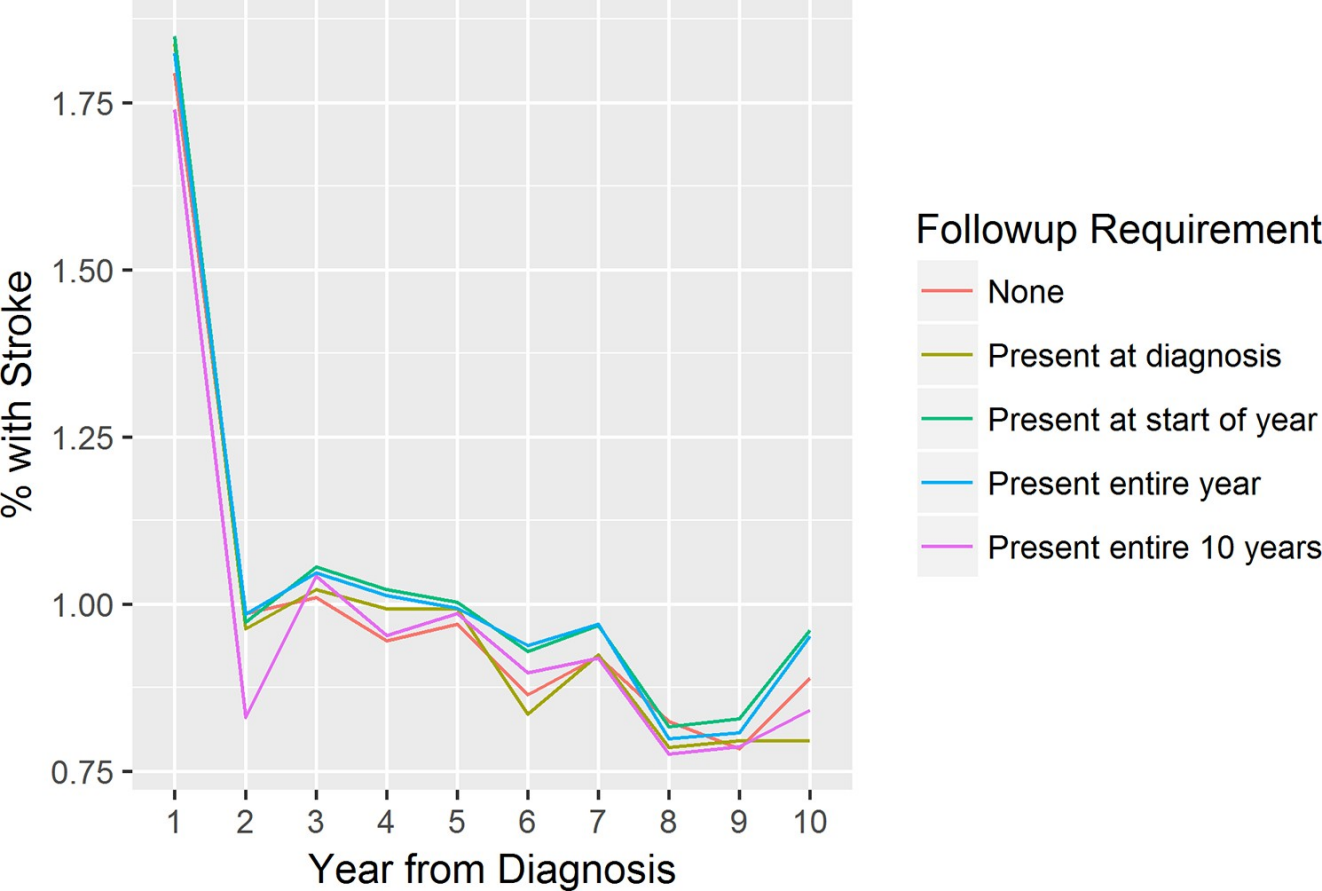
Measured incidence of stroke by year from AF diagnosis.

If follow-up is required only at diagnosis, over time the rate diverges from those who have required follow-up each year. This is illustrated in Fig 3:

Apparent differences are also observed in the stroke rates, though the story is not as clear, as seen in Fig 4:

The intent here is not to imply that the stronger follow-up requirements must be correct. This example analysis lacks a gold standard for comparison, so the optimal follow-up measure can’t be inferred from these findings. Some of the stronger follow-up requirements may themselves be subject to bias (for example, the group that is followed up continuously for 10 years may be a healthier cohort, as they may have a more stable home environment and are less likely to have experienced events like being moved to a care home). The point is that the choice of follow-up requirement has a potentially major impact on study findings—and our algorithm provides researchers with a tool to more easily implement an appropriate requirement within the context of an individual study.

## Conclusions

The method chosen to measure follow-up time in routine datasets can have a major impact on the results of analysis, as demonstrated by our example analysis of prescribing and stroke rates. Failure to robustly account for individual follow-up may lead to reporting erroneous findings. Measuring follow-up can be a complex task in large, linked datasets where individuals have complex histories and errors are present.

Standardization and reusability are key to address this complexity in an efficient manner across multiple research projects. The method described here and its implementation as a DB2 procedure have achieved this, having been used in more than 100 research projects using the SAIL Databank.

There is no perfect solution to the reality of errors in routine dataset. We believe the method described here, based on evidence from exploring the characteristics of conflicting and otherwise erroneous records, approaches a best practice solution. However, even with a “cleaned” dataset such as this, researchers need to be aware of biases, potential errors, and missing data that may still remain. This is inherent to using routinely-collected observational data, and awareness of this needs to permeate every part of the research process, including selecting appropriate study designs (and in some cases, deciding whether a question is answerable with this type of data).

There is also no one-size-fits-all approach to how information on follow-up time will be used in a study. Each study must decide this, based on appropriateness for the analysis used, recording of particular health conditions, etc. This work provides information to support enforcing follow-up requirements, rather than dictating a particular method. The illustrative examples of warfarin and stroke recording demonstrate that these choices have an impact; building on this to compare different coverage methods within a full research study, investigating the impact on the results, would be useful future work.

The current work has focused on the data held specifically by the SAIL Databank, and enabling research with this resource. Some of the issues addressed are specific to SAIL, but others are general problems faced by researchers using other data sources. Applying these methods to other data, as well as creating general tools that would work across many data sources, would be useful future steps. Being able to apply a similar coverage measure across multiple data sources would facilitate replication, which is widely viewed as key to robust science generally, and realizing the potential of routine data sources specifically [12,13].

We hope this will be one small step on the road to rapid, efficient, high-quality research in routine data, enabled by repeatable, sharable methods. Further work will focus on applying the approach here to other problems that researchers commonly face.
